# Draft genome sequence of *Collimonas* sp. strain H4R21, an effective mineral-weathering bacterial strain isolated from the beech rhizosphere

**DOI:** 10.1128/mra.00308-24

**Published:** 2024-07-22

**Authors:** Morin E., Uroz S., Kumar R., Rey M.W., Pham J., Akum F., Leveau J. H. J.

**Affiliations:** 1 Université de Lorraine, INRAE, UMR1136 « Interactions Arbres-Microorganismes », Champenoux, France; 2 INRAE, UR1138 « Biogéochimie des Ecosystèmes Forestiers », Champenoux, France; 3 Novozymes Inc., Davis, California, USA; 4 Department of Plant Pathology, University of California, Davis, California, USA; The University of Arizona, Tucson, Arizona, USA

**Keywords:** *Collimonas*, forest, nutrient-poor soil, mineral weathering, chitin, fungi

## Abstract

We present the draft genome sequence of *Collimonas* sp. strain H4R21, isolated from the rhizosphere of *Fagus sylvatica* in the forest experimental site of Montiers (France). This genome features coding capacity for plant growth promotion, such as the ability to solubilize minerals, to produce siderophores and antifungal secondary metabolites.

## ANNOUNCEMENT

In nutrient-poor soils, tree rhizosphere is typically enriched for mineral weathering bacteria (1 – 8). In such low nutrient conditions, beech trees are known to increase root exudation, which derivates can be used as carbon substrate by bacteria (9, 10). Collimonads are particularly effective at weathering (11) and share the ability to hydrolyze chitin, to produce antifungal molecules and to promote plant growth (11 – 15). To date, six species have been described (*C. anthrihumi, C. arenae, C. fungivorans, C. pratensis, C. humicola,* and *C. silvisoli*) (16 – 19). Collimonads belong to the Oxalobacteraceae family and are usually found in acidic and nutrient-poor soils (3, 5, 11). Strain H4R21 was retained for detailed analyses because of its effectiveness at weathering. It was isolated from beech rhizosphere on the Montiers site (France) (1). In October 2014, 5 g of fresh roots and adhering soil were suspended in 25 mL sterile distilled water and serial dilutions were done on 1/10 TSA medium to purify bacteria before cryo-preservation in 40% glycerol (1).

To extract DNA for sequencing, a culture on 1/10 TSA was done from the glycerol stock, and a single colony was used to inoculate 1/10 TSB medium. The culture was grown 2 days to reach late exponential phase. DNA was obtained after lysozyme (1 mg/mL) and proteinase K (1 mg/mL) treatments as described by Pospiech and Neumann (20). The library was prepared using the Nextera XT DNA library preparation kit (Illumina), following the manufacturer’s instructions. The library was sequenced as 150 × 2 bp paired reads that were generated on an Illumina MiSeq instrument (Illumina Inc.).

For all of the following programs, default parameters were used except where otherwise specified.

The sequencing resulted in 3,652,095 pairs of raw reads, which were trimmed with Trimmomatic (v0.36; (21) and assembled using SPAdes v3.9.0 (22). Gene prediction was done using prodigal v2.6.3 (23), classic RAST (24), and the NCBI Prokaryotic Genome Annotation Pipeline (PGAP) (25). RAST was used as it permits an expert and continual annotation. tRNA-scan-SE v2.0.12 (26) and Barrnap v0.9 (https://github.com/tseemann/barrnap) were used for tRNA and rRNA prediction, respectively. Complete statistics of the draft genome can be found in Table 1. A 99.9% genome completeness was estimated with BUSCO (27) compared to the Burkholderiales lineage data set.

**TABLE 1:** Genome information and statistics

Genome project information	
GenBank accession	JBANDC000000000
Bioproject no.	PRJNA1081282
SRA accession number	SRR28162545
Genome assembly statistics	
Total length (bp)	5,613,331
No. of contigs (≥500 bp)	46
N50 (bp)	281,801
L50 (contig)	8
Largest contig	531,299
GC content (%)	59.52
Genome coverage	189.97x
Genome features	
Protein-coding genes	5,144
tRNAs	47
Complete rRNAs (5S,16S,23S)	3

Digital DNA-DNA hybridization (dDDH) analysis (28) revealed that strain H4R21 scored values ranging with the type strains from 37.4% with *C. antrihumi* (DSM104040) and *C. arenae* (Ter10) to 44% with *C. pratensis* (Ter291) and *C. humicola* (RLT1W55), 45.8% with *C. silvisoli* (RXD178), and 63.1% with *C. fungivorans* (Ter331).

Homologs of proteins with a central role in the mineral-weathering ability of collimonads, including a Glucose-Methanol-Choline oxidoreductase (29) and a non-ribosomal polypeptide synthetase (NRPS) encoding for the synthesis of the siderophore malleobactin were detected (30, 31). Antismash (32) analyses revealed the presence of most of the genes encoding the production of collimomycin, an antifungal metabolite identified in *C. fungivorans* strain Ter331(13). These findings suggest that H4R21 is well equipped to survive and thrive in the rhizosphere of plants growing in nutrient-poor soils, to inhibit fungi, and to mobilize nutrients, making it a promising agent for the protection and/or promotion of plants (7, 14).

## Data Availability

The whole-genome and raw sequences are available under the accession no. JBANDC000000000 and SRR28162545 for the raw data and the genome assembly, respectively.
